# Clinical characteristics and complication risks in data‐driven clusters among Chinese community diabetes populations

**DOI:** 10.1111/1753-0407.13596

**Published:** 2024-08-13

**Authors:** Binqi Li, Zizhong Yang, Yang Liu, Xin Zhou, Weiqing Wang, Zhengnan Gao, Li Yan, Guijun Qin, Xulei Tang, Qin Wan, Lulu Chen, Zuojie Luo, Guang Ning, Weijun Gu, Yiming Mu

**Affiliations:** ^1^ School of Medicine Nankai University Tianjin China; ^2^ Department of Endocrinology the First medical center of PLA General Hospital Beijing China; ^3^ Department of Endocrinology the eighth medical center of PLA General Hospital Beijing China; ^4^ Graduate School Chinese PLA General Hospital Beijing China; ^5^ Department of Medical Oncology the Fifth Medical Center of Chinese PLA General Hospital Beijing China; ^6^ Department of Geriatrics The Second Medical Center of Chinese PLA General Hospital Beijing China; ^7^ Department of Endocrinology, Ruijin Hospital Shanghai Jiao Tong University School of Medicine Shanghai China; ^8^ Department of Endocrinology Dalian Central Hospital Dalian China; ^9^ Department of Endocrinology Zhongshan University Sun Yat‐sen Memorial Hospital Guangzhou China; ^10^ Department of Endocrinology First Affiliated Hospital of Zhengzhou University Zhengzhou China; ^11^ Department of Endocrinology First Hospital of Lanzhou University Lanzhou China; ^12^ Department of Endocrinology Southwest Medical University Affiliated Hospital Luzhou China; ^13^ Department of Endocrinology Wuhan Union Hospital, Huazhong University of Science and Technology Wuhan China; ^14^ Department of Endocrinology First Affiliated Hospital of Guangxi Medical University Nanning China

**Keywords:** Chinese community population, cluster analysis, diabetes, diabetic complication, K‐means

## Abstract

**Background:**

Novel diabetes phenotypes were proposed by the Europeans through cluster analysis, but Chinese community diabetes populations might exhibit different characteristics. This study aims to explore the clinical characteristics of novel diabetes subgroups under data‐driven analysis in Chinese community diabetes populations.

**Methods:**

We used K‐means cluster analysis in 6369 newly diagnosed diabetic patients from eight centers of the REACTION (Risk Evaluation of cAncers in Chinese diabeTic Individuals) study. The cluster analysis was performed based on age, body mass index, glycosylated hemoglobin, homeostatic modeled insulin resistance index, and homeostatic modeled pancreatic β‐cell functionality index. The clinical features were evaluated with the analysis of variance (ANOVA) and chi‐square test. Logistic regression analysis was done to compare chronic kidney disease and cardiovascular disease risks between subgroups.

**Results:**

Overall, 2063 (32.39%), 658 (10.33%), 1769 (27.78%), and 1879 (29.50%) populations were assigned to severe obesity‐related and insulin‐resistant diabetes (SOIRD), severe insulin‐deficient diabetes (SIDD), mild age‐associated diabetes mellitus (MARD), and mild insulin‐deficient diabetes (MIDD) subgroups, respectively. Individuals in the MIDD subgroup had a low risk burden equivalent to prediabetes, but with reduced insulin secretion. Individuals in the SOIRD subgroup were obese, had insulin resistance, and a high prevalence of fatty liver, tumors, family history of diabetes, and tumors. Individuals in the SIDD subgroup had severe insulin deficiency, the poorest glycemic control, and the highest prevalence of dyslipidemia and diabetic nephropathy. Individuals in MARD subgroup were the oldest, had moderate metabolic dysregulation and the highest risk of cardiovascular disease.

**Conclusion:**

The data‐driven approach to differentiating the status of new‐onset diabetes in the Chinese community was feasible. Patients in different clusters presented different characteristics and risks of complications.

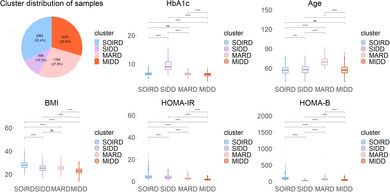

## Graphical abstract

Highlights

- The current study is the first one, to our knowledge, that clustered and typed a large sample, multicenter Chinese community‐based diabetic populations.
- This is also the first study, to our knowledge, that reported the MIDD cluster, which had a low risk burden equivalent to prediabetes, but with reduced insulin secretion.
- We explored differences in lifestyle habits and family history of disease among the various clusters of patients, which has rarely been studied previously.

## 1 INTRODUCTION

Diabetes is a chronic disease that the International Diabetes Federation (IDF) considers to be one of the fastest‐growing diseases of this century.^1^ According to the 2021 IDF report, approximately 537 million people worldwide have diabetes, which is expected to increase to 643 million by 2030 and 783 million by 2045.^2^ It was reported that 1 in 12 all‐cause deaths are attributable to diabetes.^3^ Unfortunately, despite the availability of effective therapies, the prognosis for diabetes is poor, and diabetes patients experience serious microvascular and macrovascular complications and premature death more frequently than the general population.^4^ The most commonly used definitions of diabetes are based on fasting blood glucose (FBG), posting blood glucose (PBG), and glycosylated hemoglobin (HbA1c) levels^5^; however, these criteria fail to provide therapeutic guidelines for diabetes.^6^ Indeed, considerable studies have shown that adult diabetes is a very heterogeneous metabolic disease with multiple underlying mechanisms.^7^, ^8^ Thus, a more refined classification of diabetes could identify those patients at greatest risk for complications at diagnosis and enable personalized treatment protocols.^9^

In 2018, Ahlqvist et al.^9^ used 8980 newly diagnosed diabetic populations aged 18 years or older in Sweden to classify the diabetic population into five novel subgroups based on anti‐glutamic acid decarboxylase antibody (GADA), age at diagnosis, body mass index (BMI), glycosylated hemoglobin (HbA1c), homeostatic modeled insulin resistance index (HOMA‐IR), and homeostatic modeled pancreatic β‐cell functionality index (HOMA‐β). For GADA‐positive patients, they defined it as severe autoimmune diabetes (SAID), and for GADA‐negative patients, they further categorized the patients into severe insulin‐deficient diabetes (SIDD), severe insulin‐resistant diabetes (SIRD), mild obesity‐associated diabetes (MOD), and mild age‐associated diabetes mellitus (MARD) subgroups by data‐driven cluster analysis. They found that MARD and MOD subgroups had relatively good metabolic control and relatively few diabetic complications, whereas SAID, SIDD, and SIRD subgroups had relatively poor clinical outcomes. In recent years, there have been abundant studies^6^, ^10^, ^11^, ^12^, ^13^, ^14^, ^15^, ^16^, ^17^, ^18^ utilizing data‐driven cluster analysis to divide diabetic populations into novel subgroups, and the characteristics and risk of complications of those subgroups are very similar to the study by Ahlqvist et al.^9^

At present, China has become the country with the largest number of diabetic populations.^6^ It was reported that China's diabetes populations accounted for 25% of the world's total diabetes populations^19^ Moreover, Chinese diabetic patients have distinctive characteristics from the other populations.^20^ Therefore, exploring the unique characteristics of data‐driven clusters of Chinese diabetic populations has crucial clinical guiding significance. However, the majority of China's studies have been single‐center^6^, ^16^, ^17^, ^18^ or were only on patients attending inpatient or outpatient clinics at class A tertiary hospitals.^15^, ^16^, ^17^, ^18^ Hence, the current study aims to explore the clinical characteristics of novel diabetes subgroups under data‐driven analysis in a large‐sample, multicenter Chinese community population.

## 2 METHODS

### 2.1 Database introduction

In the current study, participants were included from eight centers of the REACTION (Risk Evaluation of cAncers in Chinese diabeTic Individuals) study.^21^ Overall, 53 639 participants aged 40 years or older were investigated in the survey between March and December 2012. The research program was authorized by the Human Research of Ruijin Hospital. Before the data collection, every participant provided a written informed consent. First, we included a total of 13 364 people who met the American Diabetes Association's (ADA) criteria for diabetes (FBG ≥7.0 mmol/L or PBG ≥11.1 mmol/L or HbA1c ≥6.5%)^22^ in the current survey. Next, 2670 subjects who self‐reported having a previous diagnosis of diabetes or who were already using glucose‐lowering medications or insulin were excluded; 2864 subjects with missing important data (including, insulin, biochemical markers, body measurements, personal history of disease, or family history of disease, etc), 12 patients with extreme outliers (>5 SDs from the mean),^9^ 583 subjects with a previous diagnosis of chronic kidney disease, and 866 subjects with acute diseases that might affect the glucose metabolism (like pancreatitis, liver cirrhosis, viral hepatitis) were also excluded. Ultimately, the present study included 6369 populations with newly diagnosed diabetes.

### 2.2 Data collection

Participants were assisted by a trained staff member in a face‐to‐face conversation to complete the questionnaire, which included important information such as year of birth, personal and family medical history, current medication use, smoking and drinking habits, age, occupation, and physical activity.

After the participants removed their coats and shoes, their height, weight, waist circumference (WC), and hip circumference (HP) were measured and recorded by a trained staff. Systolic blood pressure (SBP) and diastolic blood pressure (DBP) were measured three times at five‐min intervals by the same staff member using a calibrated automatic electronic device. The average of these three measurements was taken in the statistical analysis.

Blood samples were gathered at 8–9 a.m. after an 8–10 h overnight fast. Participants without or with diabetes were tested for 75 g oral glucose tolerance test (OGTT) or 100 g steamed‐bread meal test, separately, and blood samples were gathered at 0 and 2 h. FBG and PBG were measured by the glucose oxidase method on an autoanalyzer. HbA1c was measured via the high‐performance liquid chromatography using the VARIANT II Hemoglobin Testing System. Fasting insulin was measured with chemiluminescent immunoassay. Serum creatinine (SCr) was determined by the picric acid method. Serum triglyceride (TG), total cholesterol (TC), low‐density lipoprotein cholesterol (LDL‐C), high‐density lipoprotein cholesterol (HDL‐C), aspartate transferase (AST), alanine transferase (ALT), glutamine transferase (GGT), and other biochemical indexes were determined using autoanalyzers.

The Spot morning urine samples were gathered. Urinary creatinine was determined by Jaffe's kinetic method. Urinary albumin was determined by the immunoturbidimetric assay.

### 2.3 Equations

Body mass index (BMI) (kg/m^2^) = weight (kg)/height (m)^2^. Waist‐to‐hip ratio (WHR) = WC (cm)/HC (cm). Urinary albumin–creatinine ratio (UACR) = urinary albumin (mg)/urinary creatinine (g). The estimated glomerular filtration rate (eGFR) (mL/min per 1.73 m^2^) = 175 × (SCr in mg/dL) × 1.154 × age × 0.203 × (0.742 for women) × (1.212 if African American). HOMA‐β = 20 × fasting insulin (uIU/mL)/[FBG (mmol/L)−3.5]. HOMA‐IR = fasting insulin (uIU/mL) × FBG/22:5.

### 2.4 Definitions

Hypertension was defined as SBP ≥140 mmHg or DBP ≥90 mmHg^23^ or self‐reported prior diagnosis of hypertension. Dyslipidemia was defined as TC ≥6.2 mmol/L or LDL‐C ≥ 4.1 mmol/L or TG ≥2.3 mmol/L or HDL‐C ≤1.0 mmol/L^24^ or self‐reported previous diagnosis of lipid disorders. Chronic kidney disease (CKD) was defined by eGFR <60 mL/min per 1.73 m^2^ or UACR ≥30 mg/g.^6^ Cardiovascular disease was self‐reported by patients at the time of the questionnaire survey.^25^

### 2.5 Statistical analysis

The K‐means cluster analysis was performed with the same clustering parameters (age, BMI, HbA1c, HOMA‐IR, and HOMA‐β) as the study by Ahlqvist et al.^9^ Box–Cox transformations were performed for variables not in line with normal distribution.^26^, ^27^ All values were centered to a mean value of 0 and an SD of 1. The optimal number of K‐means clusters (*K* value) was selected as 4 using silhouette methods.^9^ K‐means cluster analysis was done with randomly selected initial cluster centers (runs = 100). The cluster statistics was performed using R software (v4.2.2) with packages “MASS”, “factoextra,” and “cluster.” We named the subgroups categorized in this study based on previous research and different characteristics. To further explore the impact of clustering feature on cluster results, referring to published research, we included PBG as an additional feature on the basis of the five features from the study by Ahlavist. To compare the clustering results with each other, the clustering of six feature is directly performed using *K* = 4, and the other clustering methods were the same as the original clustering. The clustering results of the two feature sets were matched using the Sankey plot method.

Continuous variables were expressed as mean ± SD or median (25% quartile, 75% quartile), and categorical variables were presented numerically (proportionally). Differences between subgroups were compared using one‐way ANOVA (for Gaussian distribution variants), Kruskal–Wallis test (for non‐Gaussian distribution variants), and chi‐square test with multiple comparison, respectively. Binary logistic regression analysis was used to compare the risk of CKD and cardiovascular disease among the different subgroups, taking the subgroup with the lowest complication rate as the reference. Three models were also established for the logistic regression analysis to control the possible confounders. Model 1 was adjusted for age and center; model 2 was additionally adjusted for occupation, education, marriage, smoking habits, drinking habits, physical activity, and family history of diabetes; and model 3 was additionally adjusted for TG, TC, HDL‐C, LDL‐C, ALT, AST, GGT, SBP, and DBP. SPSS 24.0 (IBM, Chicago, IL) was used for this part of statistical analysis. All statistical tests were two‐sided, and *p* < 0.05 was considered statistically significant.

## 3 RESULTS

### 3.1 Cluster distribution and characteristics of the variables of clustering

**FIGURE 1:**
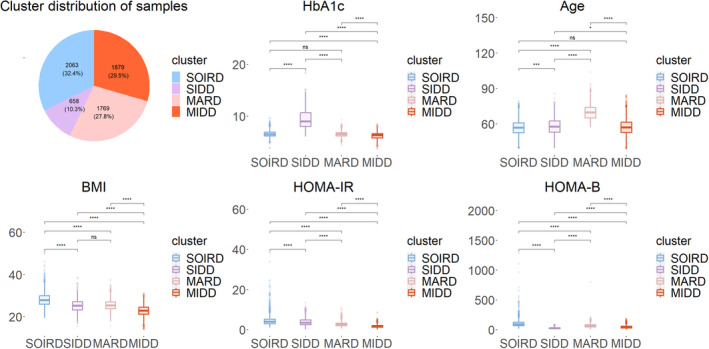
Participant distribution and cluster characteristics. The box plots represent the median and interquartile range (IQR) of the data distribution. *Significant difference between two subgroups in the multiple comparisons of analysis of variance (ANOVA) test (age, BMI, HbA1c) and Kruskal–Wallis test (HOMA‐IR，HOMA‐β). BMI, body mass index; HbA1c, hemoglobin A1c; HOMA‐IR, homoeostatic model assessment estimates of insulin resistance; HOMA‐β, homoeostatic model assessment estimates of β‐cell function; MARD, mild age‐associated diabetes mellitus; MIDD, mild insulin‐deficient diabetes; SIDD, severe insulin‐deficient diabetes; SOIRD, severe obesity‐related and insulin‐resistant diabetes. ns: *p* ≥ 0.05; *p < 0.05; **p < 0.01; ***p < 0.001; ****p < 0.0001; ns, non significance.

The subjects were classified into four diabetes subgroups based on five variables (Figure 1 and Table 1). Overall, 2063 (32.39%), 658 (10.33%), 1769 (27.78%), and 1879 (29.50%) patients were assigned to sever obesity related and insulin resistant diabetes (SOIRD), SIDD, MARD, and mild insulin‐deficient diabetes (MIDD) subgroups, respectively.

**TABLE 1:** Characteristics of the clustered variables in Chinese community diabetes populations.

Variables	Total	SOIRD	SIDD	MARD	MIDD	*p* value
Number (%)	6369	2063 (32.39)	658 (10.33)	1769 (27.78)	1879 (29.50)	
Age of diagnosis	60.62 ± 9.11	56.60 ± 6.82	58.09 ± 7.92	69.98 ± 6.43	57.13 ± 7.41	<0.001
BMI (kg/m^2^)	25.61 ± 3.58	28.18 ± 3.30	25.44 ± 3.17	25.58 ± 2.65	22.87 ± 2.58	<0.001
HbA1c (%)	6.78 ± 1.27	6.58 ± 0.62	9.53 ± 1.98	6.55 ± 0.54	6.23 ± 0.58	<0.001
HOMA‐IR	2.67 (1.83,3.90)	3.93 (3.00,5.23)	3.44 (2.49, 4.80)	2.54 (1.98, 3.26)	1.62 (1.19, 2.16)	<0.001
HOMA‐β	58.09 (37.31, 86.67)	90.30 (66.67, 122.85)	23.41 (14.26, 32.95)	51.74 (45.84, 81.30)	41.86 (28.65, 57.56)	<0.001

*Note*: Continuous variables were expressed as mean ± SD or median (25% quartile, 75% quartile), and categorical variables were presented numerically (proportionally).

Abbreviations: BMI, body mass index; HbA1c, glycosylated hemoglobin; HOMA‐IR, homoeostatic modeled insulin resistance index; HOMA‐β, homeostatic modeled pancreatic β‐cell functionality index; MARD, mild age‐associated diabetes mellitus; MIDD, mild insulin‐deficient diabetes; SIDD, severe insulin‐deficient diabetes; SOIRD, severe obesity‐related and insulin‐resistant diabetes.

The mean age (SD) of the 6369 newly diagnosed subjects with diabetes was 60.62 years (9.11), the mean BMI (SD) was 25.61 (3.58), the mean HbA1c (SD) was 6.78 (1.27), the median HOMA‐IR (Q1–Q3) was 2.67 (1.83–3.90), and the median HOMA‐β (Q1–Q3) was 58.09 (37.31–86.67).

Participants assigned to the SOIRD subgroup had the highest BMI (mean: 28.18 kg/m^2^), the highest HOMA‐β (median: 90.30) and the highest HOMA‐IR (median: 3.93) values, the youngest age at diagnosis (mean: 56.60 years old), and moderate HbA1c (mean: 6.58%). Participants assigned to the SIDD subgroup were characterized by the highest HbA1c (mean: 9.53%) and lowest HOMA‐β (median: 23.41) values; meanwhile, this cluster had relatively high HOMA‐IR (median: 3.44) value and moderate BMI (mean: 25.44) and age (mean: 58.09). Participants assigned to the MARD subgroup had the oldest age of diagnosis (mean: 69.98), moderate BMI (mean: 25.58) and HbA1c (mean: 6.55), and moderate insulin release and insulin resistance status (median HOMA‐β: 51.74, median HOMA‐IR: 2.54). Participants assigned to the MIDD subgroup manifested the lowest BMI (mean: 22.87), lowest HbA1c (mean: 6.23), and lowest HOMA‐IR (median 1.62) value; besides, they had the moderate age of diagnosis (mean: 57.13) and HOMA‐β (median: 41.86).

### 3.2 Differences in anthropometric and biochemical indicators between subgroups

**FIGURE 2:**
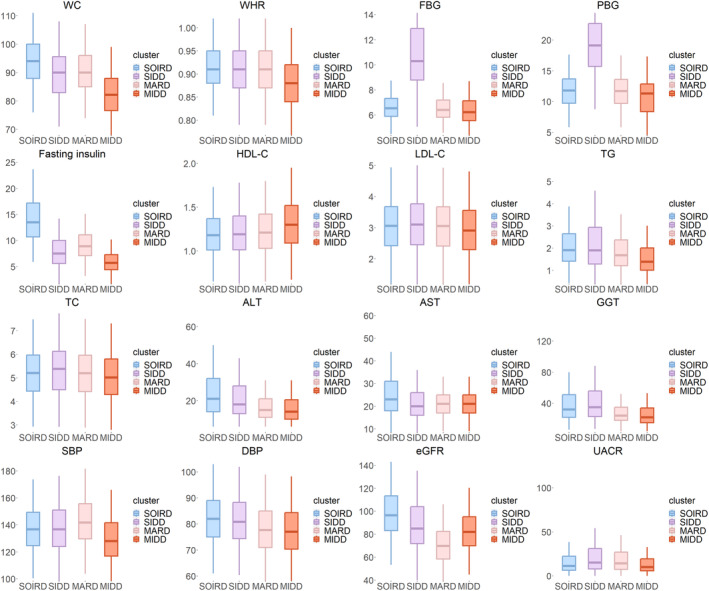
Box plot of major clinical indicators. The box plots represent the median and interquartile range (IQR) of the data distribution. ALT, alanine transferase; AST, aspartate transferase; DBP, diastolic blood pressure; eGFR, estimated glomerular filtration rate; FBG, fasting blood glucose; GGT, glutamine transferase; HDL‐C, high‐density lipoprotein cholesterol; LDL‐C, low‐density lipoprotein cholesterol; MARD, mild age‐associated diabetes mellitus; MIDD, mild insulin‐deficient diabetes; PBG, posting blood glucose; SBP, systolic blood pressure; SIDD, severe insulin‐deficient diabetes; SOIRD, severe obesity‐related and insulin‐resistant diabetes; TC, total cholesterol; TG, triglyceride; WC, waist circumference; WHR, waist‐to‐hip ratio.

In Table 2 and Figure 2, participants assigned to the SOIRD subgroup were characterized by the largest WC (mean 93.97), highest fasting insulin levels (median: 13.50), lowest HDL‐C (median: 1.18), highest TG (median: 1.91), highest ALT (median: 21), AST (median: 23), and DBP (median: 82). Participants assigned to the SIDD subgroup had the highest FBG (median: 10.30), PBG (median: 19.13), LDL‐C (median: 3.10), TC (median: 5.38), GGT (median: 32), and UACR (median: 15.56), as well as the highest prevalence rate of dyslipidemia (64%) and CKD (28.59%). Participants assigned to the MARD subgroup manifested the highest SBP (median 142), the highest proportion of hypertensive patients (70.1%), and the lowest eGFR (mean 83.59). Participants assigned to the MIDD subgroup showed the lowest FBG (median: 6.23), PBG (median: 11.31), LDL‐C (median: 2.91), TG (median: 1.38), TC (median: 5.02), ALT (median: 14), AST (median: 21), SBP (median: 128), DBP (median: 77), and UACR (median: 10.28), as well as the lowest percentage of patients with dyslipidemia (43%), hypertension (38.6%), and CKD (14%), while HDL‐C (median: 1.30) and eGFR (mean: 94.15) were the highest.

**TABLE 2:** Characteristics of the physical examination and laboratory test among newly diagnosed Chinese community diabetes populations in different subgroups.

Variables	Total	SOIRD	SIDD	MARD	MIDD	*p* value
WC	89.07 ± 9.92	93.97 ± 9.10	89.34 ± 9.15	90.41 ± 8.49	82.32 ± 8.46	<0.001
WHR	0.90 ± 0.67	0.91 ± 0.61	0.91 ± 0.62	0.91 ± 0.64	0.88 ± 0.71	<0.001
FBG	6.57 (5.84, 7.53)	6.54 (5.90, 7.33)	10.30 (8.79, 12.91)	6.40 (5.83, 7.19)	6.23 (5.56, 7.13)	<0.001
PBG	11.85 (9.60, 14.15)	11.80 (9.74, 13.70)	19.13 (15.69, 22.69)	11.70 (9.69, 13.60)	11.31 (8.35, 12.87)	<0.001
Fasting insulin	8.80 (6.20, 12.40)	13.50 (10.70, 17.20)	7.50 (5.58, 10.00)	8.90 (7.10, 11.10)	5.70 (4.40, 7.30)	<0.001
HDL‐C	1.22 (1.04, 1.43)	1.18 (1.01, 1.37)	1.19 (1.01, 1.40)	1.21 (1.03, 1.42)	1.30 (1.09, 1.52)	<0.001
LDL‐C	3.02 (2.39, 3.65)	3.06 (2.42, 3.68)	3.10 (2.45, 3.77)	3.05 (2.41, 3.67)	2.91 (2.29, 3.56)	<0.001
TG	1.69 (1.19, 2.41)	1.91 (1.41, 2.65)	1.90 (1.28, 2.94)	1.68 (1.21, 2.38)	1.38 (1.00, 2.01)	<0.001
TC	5.17 (4.39, 5.95)	5.21 (4.44, 5.97)	5.38 (4.50, 6.14)	5.20 (4.42, 5.96)	5.02 (4.29, 5.81)	<0.001
Dyslipidemia, *n* (%)						<0.001
Yes	3400 (53.4)	1245 (60.3)	421 (64)	926 (52.3)	808 (43)	
No	2969 (46.6)	818 (39.7)	237 (36)	843 (47.7)	1071 (57)	
ALT	17 (12, 25)	21 (14, 32)	18 (13, 28)	15 (11, 21)	14 (10, 21)	<0.001
AST	21 (17, 27)	23 (18, 31)	20 (16, 26)	21 (17, 25)	21 (17, 25)	<0.001
GGT	26 (18, 42)	32 (22, 51)	35 (23, 56)	24 (18, 35.5)	22 (15, 34)	<0.001
SBP	136 (123,149)	137 (125,149)	137 (124,151)	142 (130,156)	128 (117,142)	<0.001
DBP	79 (72, 87)	82 (75, 89)	81 (74, 88)	78 (71, 85)	77 (70, 84)	<0.001
Hypertension, *n* (%)						<0.001
Yes	3482 (54.7)	1176 (57)	341 (51.8)	1240 (70.1)	725 (38.6)	
No	2887 (45.3)	887 (43)	317 (48.2)	529 (29.9)	1154 (61.4)	
eGFR	89.88 ± 19.34	91.26 ± 19.15	90.33 ± 19.60	83.59 ± 19.03	94.15 ± 18.25	<0.001
UACR	12.25 (6.96, 23.93)	11.86 (6.71, 23.05)	15.56 (8.37, 31.71)	14.69 (7.92, 27.26)	10.28 (6.19, 19.66)	<0.001
Chronic kidney disease, *n* (%)						<0.001
Yes	1340 (21.04)	417 (20.21)	188 (28.59)	472 (26.67)	263 (14.0)	
No	5029 (78.96)	1646 (79.79)	470 (71.41)	1297 (73.33)	1616 (86)	

*Note*: Continuous variables were expressed as mean ± SD or median (25% quartile, 75% quartile), and categorical variables were presented numerically (proportionally).

Abbreviations: ALT, alanine transferase; AST, aspartate transferase; BMI, body mass index; DBP, diastolic blood pressure; eGFR, estimated glomerular filtration rate; FBG, fasting blood glucose; GGT, glutamine transferase; HbA1c, hemoglobin A1c; HDL‐C, high‐density lipoprotein cholesterol; HOMA‐β, homeostatic modeled pancreatic β‐cell functionality index; HOMA‐IR, homeostatic modeled insulin resistance index; LDL‐C, low‐density lipoprotein cholesterol; MARD, mild age‐associated diabetes mellitus; MIDD, mild insulin‐deficient diabetes; PBG, posting blood glucose; SBP, systolic blood pressure; SIDD, severe insulin‐deficient diabetes; SOIRD, severe obesity‐related and insulin‐resistant diabetes; TC, total cholesterol; TG, triglyceride; UACR, urinary albumin–creatinine ratio; WC, waist circumference; WHR, waist‐to‐hip ratio.

### 3.3 Differences in lifestyle habits, personal, and family history of disease between clusters

In Table 3, SIDD (47.87%) and MIDD (40.18%) clusters had a relatively high proportion of males, and SOIRD cluster had the lowest percentage of males (29.76%). Participants assigned to SIDD were more likely to have smoking and drinking habits than the other clusters. Family history of diabetes was most common in the SOIRD subgroup (21.93%) and least common in the MARD subgroup (10.25%). Patients in MARD subgroup (12.83%) had the highest prevalence of cardiovascular diseases. Self‐reported fatty liver was most common in patients in SOIRD subgroup (62.34%). Self‐reported retinopathy was most common in patients in the MARD subgroup (0.79%). Patients in the SOIRD subgroup reported the most tumor history (3.11%) and family history of tumors (15.29%) compared with those in the other subgroup.

**TABLE 3:** Characteristics of the questionnaire information in Chinese community diabetes populations in different subgroups.

Variable	Total	SOIRD	SIDD	MARD	MIDD	*p* value
Sex, *n* (%)						<0.001
Male	2323 (36.47)	614 (29.76)	315 (47.87)	639 (36.12)	755 (40.18)	
Female	4046 (63.53)	1449 (70.24)	343 (52.13)	1130 (63.88)	1124 (59.82)	
Smoking, *n* (%)						<0.001
Never	5348 (83.97)	1768 (85.70)	507 (77.05)	1584 (89.54)	1489 (79.24)	
Occasional	177 (2.78)	57 (2.76)	21 (3.19)	38 (2.15)	61 (3.25)	
Frequently	844 (13.25)	238 (11.54)	130 (19.76)	147 (8.31)	329 (17.51)	
Drinking, *n* (%)						<0.001
Never	4672 (73.35)	1541 (74.70)	461 (70.06)	1386 (78.35)	1284 (68.33)	
Occasional	1152 (18.09)	365 (17.69)	116 (17.63)	271 (15.32)	400 (21.29)	
Frequently	545 (8.56)	157 (7.61)	81 (12.31)	112 (6.33)	195 (10.38)	
Physical activity level						<0.001
Low	5195 (81.57)	1707 (82.74)	549 (83.43)	1451 (82.03)	1488 (79.19)	
Moderate	802 (12.59)	224 (10.86)	80 (12.16)	250 (14.13)	248 (13.20)	
High	372 (5.84)	132 (6.40)	29 (4.41)	68 (3.84)	143 (7.61)	
Family history of diabetes, *n* (%)						<0.001
Yes	1145 (17.98)	452 (21.93)	137 (20.86)	181 (10.25)	375 (19.97)	
No	5224 (82.02)	1611 (78.07)	521 (79.14)	1588 (89.75)	1504 (80.03)	
Self‐reported cardiovascular disease, *n* (%)						<0.001
Yes	447 (7.02)	122 (5.91)	28 (4.26)	227 (12.83)	70 (3.73)	
No	5922 (92.98)	1941 (94.09)	630 (95.74)	1542 (87.17)	1809 (96.27)	
Self‐reported fatty liver, *n* (%)						<0.001
Yes	2423 (38.04)	1286 (62.34)	301 (45.74)	590 (33.35)	246 (13.09)	
No	3946 (61.96)	777 (37.66)	357 (54.26)	1179 (66.65)	1633 (86.91)	
Self‐reported retinopathy, *n* (%)						<0.001
Yes	29 (0.46)	4 (0.19)	4 (0.61)	14 (0.79)	7 (0.37)	
No	6340 (99.54)	2059 (99.81)	654 (99.39)	1755 (99.21)	1872 (99.63)	
Self‐reported tumor history, *n* (%)						<0.001
Yes	180 (2.83)	64 (3.11)	17 (2.60)	43 (2.44)	56 (2.99)	
No	6189 (98.17)	1999 (96.89)	641 (97.40)	1726 (97.56)	1823 (97.01)	
Family history of tumor, *n* (%)						<0.001
Yes	813 (12.76)	315 (15.29)	68 (10.28)	177 (10.02)	253 (13.44)	
No	5556 (87.24)	1748 (84.71)	590 (89.72)	1592 (89.98)	1626 (86.56)	

*Note*: Categorical variables were presented numerically (proportionally).

Abbreviations: MARD, mild age‐associated diabetes mellitus; MIDD, mild insulin‐deficient diabetes; SIDD, severe insulin‐deficient diabetes; SOIRD, severe obesity‐related and insulin‐resistant diabetes.

### 3.4 Risk of CKD and cardiovascular disease by subgroups

Table 4 shows the results of the logistic regression analysis. The MIDD subgroups with the lowest prevalence of CKD (14.0%) was used as the reference group. In model 3, after adjusting for the confounding factors, populations in SIDD (odds ratio [OR], 2.240; 95% confidence interval [CI], 1.755–2.860; *p* < 0.001), SOIRD (OR, 1.383; 95% CI, 1.138–1.681; *p* = 0.001), and MARD (OR, 1.619; 95% CI, 1.328–1.973; *p* < 0.001) subgroups all showed higher risks of CKD compared with MIDD subgroup, and the differences were all statistically significant. Besides, The MIDD cluster with the lowest prevalence of cardiovascular disease (3.73%) was used as the reference group. In model 3, after adjusting for the confounding factors, populations in SOIRD (OR, 1.573; 95% CI, 1.105–2.139; *p* = 0.011) and MARD (OR, 1.564; 95% CI, 1.120–2.185; *p* = 0.009) subgroups showed higher risks of cardiovascular disease compared with MIDD subgroup, and the differences were all statistically significant; however, there was no statistically significant difference in the risk of cardiovascular disease between the SIDD and MIDD subgroups (*p* = 0.894).

**TABLE 4:** Association between diabetes‐related complications and subgroups.

	*N*	*n* (%)	OR (95% CI)							
			Unadjusted	*p* value	Model 1	*p* value	Model 2	*p* value	Model 3	*p* value
Chronic kidney disease										
MIDD	1879	263 (14.0)	Reference		Reference		Reference		Reference	
SIDD	658	188 (28.59)	2.460 (1.977, 3.062)	<0.001	2.799 (2.231, 3.511)	<0.001	2.707 (2.144, 3.418)	<0.001	2.240 (1.755, 2.860)	<0.001
SOIRD	2063	417 (20.21)	1.557 (1.309, 1.851)	<0.001	1.671 (1.398, 1.997)	<0.001	1.691 (1.409, 2.030)	<0.001	1.383 (1.138, 1.681)	0.001
MARD	1769	472 (26.67)	2.235 (1.882, 2.654)	<0.001	2.183 (1.829, 2.606)	<0.001	2.016 (1.668, 2.435)	<0.001	1.619 (1.328, 1.973)	<0.001
Cardiovascular disease										
MIDD	1879	70 (3.73)	Reference		Reference		Reference		Reference	
SIDD	658	28 (4.26)	1.149 (0.734, 1.797)	0.544	1.090 (0.695, 1.709)	0.707	1.004 (0.630, 1.600)	0.986	0.968 (0.598, 1.566)	0.894
SOIRD	2063	122 (5.91)	1.624 (1.202, 2.194)	0.002	1.549 (1.144, 2.098)	0.005	1.533 (1.125, 2.089)	0.007	1.537 (1.105, 2.139)	0.011
MARD	1769	227 (12.83)	3.804 (2.886, 5.016)	<0.001	3.606 (2.728, 4.766)	<0.001	2.682 (1.997, 3.603)	<0.001	1.564 (1.120, 2.185)	0.009

*Note*: Model 1 was adjusted for age and center; model 2 was additionally adjusted for occupation, education, marriage, smoking habits, drinking habits, physical activity, and family history of diabetes; and model 3 was additionally adjusted for TG, TC, HDLC, LDLC, ALT, AST, GGT, SBP, and DBP.

Abbreviations: ALT, alanine transferase; AST, aspartate transferase; DBP, diastolic blood pressure; GGT, glutamine transferase; HDL‐C, high‐density lipoprotein cholesterol; LDL‐C, low‐density lipoprotein cholesterol; MARD, mild age‐associated diabetes mellitus; MIDD, mild insulin‐deficient diabetes; SBP, systolic blood pressure; SIDD, severe insulin‐deficient diabetes; SOIRD, severe obesity‐related and insulin‐resistant diabetes; TC, total cholesterol; TG, triglyceride.

### 3.5 Difference between the original cluster features and features with PBG

Figure S1 showed that adding or not adding PBG as a clustering variable resulted in a difference in clustering result in only about 3.6% (230/6369) of the individuals, and the Table S1 similarly showed that the characteristics of the typologies obtained by adding PBG as a clustering variable are essentially similar to those obtained from analyses conducted in the present study.

## 4 DISCUSSION

Unlike the Ahlqvist research, the present study identified a specific cluster, SOIRD, which had appeared in European non‐newly diagnosed diabetic populations^28^ and in community diabetes population older than 40 years in Shanghai, China.^6^ SOIRD subgroup had the highest BMI, HOMA‐IR and HOMA‐β, and the second highest HbA1c (after SIDD), which combined the characteristics of SIRD and MOD subgroups from previous studies, that is, obesity with insulin resistance. While the study of the Shanghai community diabetes population added PBG to the variables used in Ahlqvist's study, the current study used only the five variables used by Ahlqvist's study, and also found the SOIRD cluster. Besides, we additionally attempted to use PBG as a variable for clustering, and the Sankey plot in Figure S1 showed that adding or not adding PBG as a clustering variable resulted in a difference in clustering result in only about 3.6% of the individuals, and Table S1 similarly showed that the characteristics of the typologies obtained by adding PBG as a clustering variable are essentially similar to those obtained from analyses conducted in the present study. A study of 19 084 Indian diabetic populations using eight variables (age at diagnosis, BMI, WC, HbA1c, TG, HDL‐C, fasting C‐peptide, poststimulation C‐peptide) also obtained insulin resistant and obesity related diabetes (IROD), a subgroup with the highest HOMA‐β, HOMA‐IR, and BMI (similar to our SOIRD). There is still academic controversy about the variables used to generate patient subtypes.^4^, ^29^ In fact, the OGTT and postprandial islet function test are still expensive and inconvenient for a routine exam; our current clustering method could use fewer variables to obtain the comparable clusters result as those in the Shanghai community diabetic populations and Indian diabetic populations, which would undoubtedly save time costs and economic costs. The MARD subgroup tended to be the most prevalent in previous studies,^4^ whereas the SOIRD cluster was the most prevalent in the current study, which might be attributed to differences in ethnicity and differences between inpatients and patients in the community; in addition, the study populations in our study were residents over 40 years of age (the common age of onset of diabetes), whereas most of the previous studies have focused on adults over 18 years old; the difference in age could also lead to a difference in category.

The SOIRD subgroup had the largest WC. Unlike BMI, WC is more responsive to visceral adipose tissue mass,^30^ and a previous study^31^ has found that the amount of visceral adipose tissue is higher in Asians than in Westerners at similar BMI, leading to greater insulin resistance in Asian patients with diabetes. We also observed the lowest HDL‐C and highest TG in the SOIRD cluster, and that a strong association between dyslipidemia and insulin resistance has long been established.^32^, ^33^ Besides, the SOIRD cluster had the highest ALT and AST, and highest prevalence of self‐reported fatty liver, which is consistent with previous studies.^6^, ^15^ Moreover, benefiting from the detailed questionnaire, the present study also innovatively found that the SOIRD category had the highest rates of tumor, family history of diabetes, and family history of tumors. We hypothesized that unhealthy lifestyle habits among family members influence each other, such as poor dietary habits causing obesity and insufficient exercise^34^ and obesity^31^ leading to insulin resistance, therefore, diabetic patients in the SOIRD cluster exhibit familial aggregation. However, it is unfortunate that the causal relationship between poor lifestyle and the SOIRD subgroup is not clear in this study, and we hope that prospective cohort studies will be conducted in the future to clarify whether changes in poor dietary habits and sensible exercise could improve insulin resistance and glucose metabolism abnormalities in diabetes populations with SOIRD subgroup typing. In addition, there is evidence that obesity drives morphological and functional changes in cancer cells through complex interactions within the tumor microenvironment,^35^ while hyperinsulinemia promotes tumor development^36^; on the other hand, insulin resistance is closely associated with visceral fat dysfunction and systemic inflammation, both of which are conducive to the establishment of a protumorigenic milieu,^36^ and the above could explain the high tumor disease history of the SOIRD subgroup.

In the current study, a new diabetes subgroup, MIDD, was identified, which had lower HOMA‐β (only higher than the SIDD subgroup but significantly lower than the other subgroups), suggesting insufficient insulin secretion. In addition, HbA1c and FBG in the MIDD subgroup were the lowest, with the medians being in the prediabetic category, whereas the median PBG met the diagnostic criteria for diabetes, which is typical of Chinese diabetes populations, as the diet of the Chinese population is characterized by a predominance of high carbohydrates.^37^ The MIDD subgroup had the lowest BMI and WC, both of which were in the normal range, and other biochemical indices were better than those of the other subgroups, suggesting that metabolic control was relatively good in this subgroup. In fact, compared with Caucasian populations, Asian populations with diabetes tend to be thinner but have more impaired pancreatic function^38^ because Asians have an innate deficiency in insulin secretion capacity.^39^ The present study is of great clinical significance in identifying MIDD as a subgroup of diabetes among Chinese population, because this type of diabetic population tends to ignore their condition due to mild or no symptoms; however, their pancreatic islet function has already been damaged, so this diabetic population is advised to have regular medical checkups and to take reasonable measures for glycemic control in order to avoid further impairment of the pancreatic islet function.

In agreement with previous studies,^6^, ^9^, ^10^, ^15^, ^16^, ^17^ the current study also found that the SIDD subgroup had the lowest HOMA‐β, suggesting that pancreatic islet function had been severely damaged, and had the highest HbA1c, FBG and PBG, suggesting poor glycemic control. Besides, we found the highest prevalence of dyslipidemia in this subgroup, indicating poorer metabolic control. Previous studies have shown that newly diagnosed Chinese diabetic populations with combined hyperlipidemia exhibit decreased insulin secretion rather than impaired insulin sensitivity,^40^ which might be due to dyslipidemia leading to elevated circulating free fatty acid levels, resulting in impaired β‐cell function.^40^ There is evidence that antihyperlipidemic drugs improve β‐cell function in diabetic populations.^41^ Given the highest prevalence of dyslipidemia in the SIDD subgroup of this study, we consider that stricter lipid control in this type of populations might be a new therapeutic idea. We also found that the SIDD subgroup had the highest UACR, and previous studies^6^, ^18^ have similarly found that this subgroup has the highest risk of albuminuria. In addition, logistic regression analysis showed that despite adjusting for confounders, the risk of CKD was still highest in the SIDD subgroup, which was 2.24 times higher than in the lowest subgroup. The causal relationship between abnormal β‐cell function and the development of albuminuria has been demonstrated.^42^ Furthermore, we found that the SIDD subgroup had the largest percentage of smokers and drinkers, followed closely by the MIDD subgroup. Numerous population‐based studies^43^, ^44^, ^45^, ^46^ have shown a strong association between smoking and alcohol consumption and impaired β‐cell function. Studies using rodent models have shown that nicotine exposure could mediate β‐cell dysfunction, pancreatic β‐cell apoptosis, and β‐cell mass loss through mitochondrial or death receptor pathways.^47^ Studies in rats have also shown that chronic high‐dose alcohol intake could lead to altered insulin, messenger RNA (mRNA) gene expression, and impaired pancreatic β‐cell function.^48^ This might also explain the high percentage of men in the SIDD and MIDD subgroups, as men are more likely to be smokers and drinkers and such habits decrease insulin secretion. We recommend education on smoking and alcohol cessation for diabetic populations classified as SIDD or MIDD subgroups to avoid further decline in pancreatic β‐cell functions.

Consistent with previous studies,^4^, ^6^, ^9^, ^11^, ^14^, ^15^, ^16^, ^18^, ^28^ the MARD subgroup in the current study had the oldest age and moderate metabolic dysregulation, and the lowest prevalence of family history of diabetes.^6^ The MARD subgroup had the highest prevalence of hypertension and the highest SBP; however, it is noteworthy that DBP was lower than average in this subgroup (only slightly higher than that in the SIDD cluster), suggesting that populations in this subgroup should be alerted not only to the risk of hypertension but also to the risk of increased arterial stiffness represented by a high pulse pressure gap.^49^ In logistic regression analyses, we found that despite adjusting for numerous biochemical and lifestyle confounders, the MARD subgroup had the highest risk of cardiovascular disease, which was 1.564 times higher than that of the lowest subgroup. Advanced age is a well‐known risk factor for cardiovascular disease^50^; therefore even though patients in the MARD subgroup did not have the worst glycemic levels and metabolic control, the risk of developing major cardiovascular disease was the highest. Interestingly, in logistic regression analyses, the SOIRD subgroup also had a high cardiovascular risk, which was 1.537 times that of the lowest subgroup. It has been demonstrated that the degree of insulin resistance could independently influence macrovascular complications of diabetes^51^; consequently, even after adjusting for confounders, the risk of cardiovascular disease was high in the SOIRD subgroup, although age was lowest in this subgroup.

The current study is the first one that clustered and typed a large sample, multicenter Chinese community diabetes patients. The current study population is more generally representative than patients attending hospitals. In addition, we have detailed information on possible confounding factors. Furthermore, benefiting from the detailed questionnaire in the present study, we could explore differences in lifestyle habits and family history of disease among the various subgroups of populations, which has rarely been explored in previous studies. This also allows us to provide more individualized advice to middle‐aged and elderly Chinese community diabetic populations; for instance, SOIRD populaions should change their poor dietary habits and exercise more, SIDD and MIDD populations need to reduce smoking and drinking habits, and MARD populations should focus not only on blood glucose but also on the risk of hypertension and atherosclerosis. However, the current study still has some limitations. First, GADA were not measured or included in the cluster analysis in the present study. However, the prevalence of GADA‐positive diabetes among Chinese adults with diabetes is likely to be less than 5.9%^6^, ^10^ and might be even lower in population‐based screening because GADA‐positive diabetic populations are usually diagnosed before they are captured by screening owing to their acute diabetic complications.^10^ Second, the age of the subjects in the current study was 40 years and older; however, this is the common age for the onset of diabetes.^52^ There should be studies clustering and typing community diabetes populations in broader age groups. Third, the present study is a cross‐sectional study, and previous investigation^53^ reported that the subgroup typing of diabetic populations could be transformed with the course of the disease; however, in the present study, it remains inaccessible to learn whether the current subgroups transform with the disease progression. In addition, without follow‐up data, we were unable to further investigate whether the clustering method in the present study was superior to prediction methods based on baseline variables or supervised machine learning methods in terms of its ability to predict patients' future complications and disease severity. Moreover, due to the lack of follow‐up data on these patients newly diagnosed with diabetes in the present epidemiologic survey, we do not know the effect of glucose‐lowering drugs on complications in these subgroups of diabetic populations and the proportion of death from complications in different subgroups of populations. In order to expand our study and better assist in the clinical management of diabetes mellitus, long‐term follow‐up studies are essential to understand illness progression and therapy response.

## 5 CONCLUSIONS

In the current study, the data‐driven approach to differentiating the status of new‐onset diabetes in the Chinese community was reproducible, and the distribution of patients was similar, but not identical, to that of the study by Ahlqvist et al. We first reported the MIDD subgroup, which had a low risk burden equivalent to prediabetes, but with reduced insulin secretion. The SOIRD subgroup was characterized by obesity and insulin resistance and had a high prevalence of fatty liver, tumors, family history of diabetes, and family history of tumors. The SIDD subgroup had severe insulin deficiency, the poorest glycemic control, and the highest prevalence of dyslipidemia and CKD. The MARD subgroup in the current study had the oldest age and moderate metabolic dysregulation, and the highest risk of cardiovascular disease and hypertension. The findings of the current study could contribute to improve early prevention and targeted treatment of diabetes in Chinese community populations.

## AUTHOR CONTRIBUTIONS

All authors have read and approved the final manuscript. Binqi Li and Yang Liu contributed to the conception and design of the study. Binqi Li, Yang Liu, Xin Zhou, Weiqing Wang, Zhengnan Gao, Li Yan, Guijun Qin, Xulei Tang, Qin Wan, Lulu Chen, Zuojie Luo, Guang Ning, Weijun Gu and Yiming Mu recruited the subjects and supervised the study. Binqi Li and Zizhong Yang analyzed the data. Binqi Li wrote the initial draft of the paper. Yiming Mu, Weijun Gu, Yang Liu, Zizhong Yang, and Binqi Li contributed to the manuscript's writing, reviewing, and revising. Binqi Li, Zizhong Yang, and Yang Liu have contributed equally to this work and share first authorship.

## FUNDING INFORMATION

The study is supported by Beijing Municipal Science and Technology Commission Project (Z201100005520014), PLA General Hospital Youth Independent Innovation Science Fund project (22QNFC052).

## CONFLICT OF INTEREST STATEMENT

Weiqing Wang, Lulu Chen, and Guang Ning are Editorial Board members of *Journal of Diabetes* and co‐authors of this article. To minimize bias, they were excluded from all editorial decision‐making related to the acceptance of this article for publication.

## Supporting information


Figure S1.

Table S1.

## Data Availability

The data sets are not freely available due to protection of participants' privacy.
