# Supplementary material for: Clinical characteristics and complication risks in data‐driven clusters among Chinese community diabetes populations
Source: J Diabetes. 2024 Aug 13;16(8):e13596. doi: 10.1111/1753-0407.13596 (PMC11320751; doi:10.1111/1753-0407.13596)
Supplement: Supplementary file 1 — Figure S1. [file JDB-16-e13596-s001.docx]

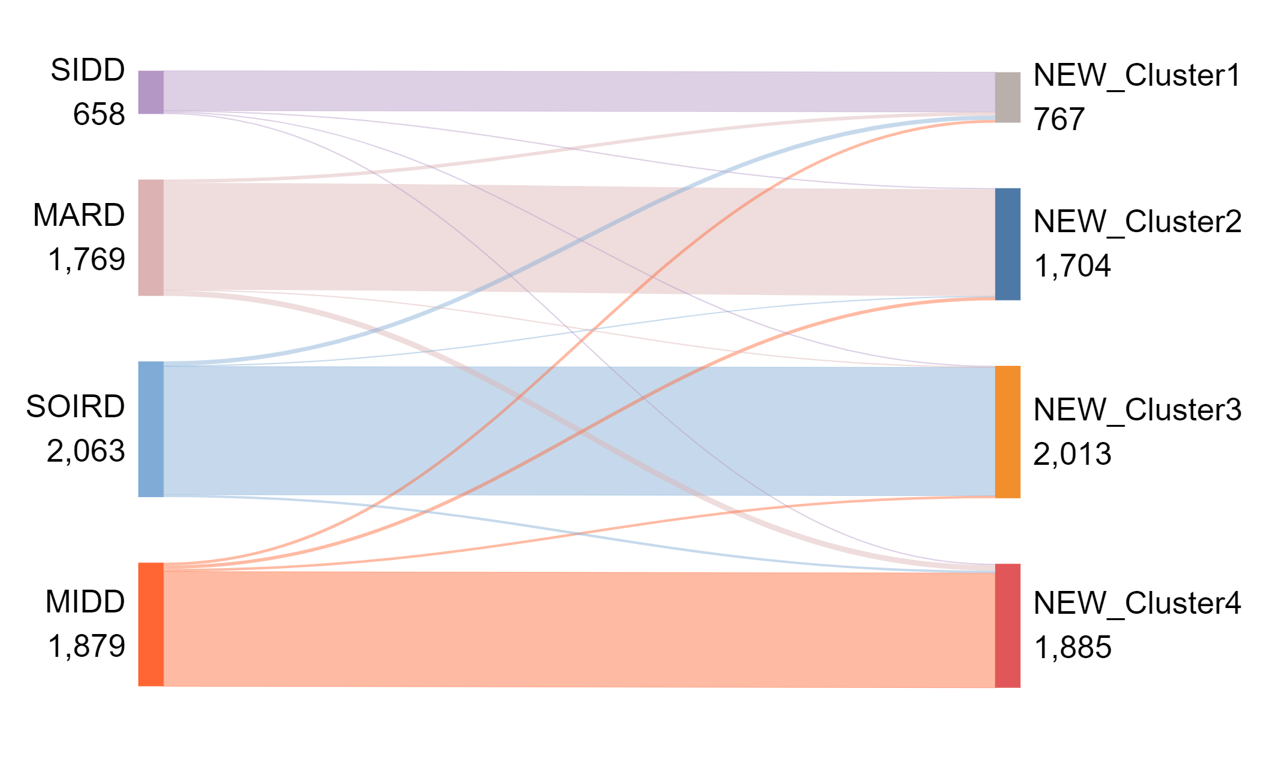


**Supplementary figure1 Sankey plot between the original cluster**

**features and features with PBG**

Abbreviations: MARD, mild age-related diabetes; MIDD, mild

insulin-deficient diabetes; SIDD, severe insulin-deficient diabetes; PBG,

posting blood glucose; SOIRD, severe obesity-related and

insulin-resistant diabetes

Note: The number under each category represents the number of

individuals in that category
