# Supplementary material for: Clinical characteristics and complication risks in data‐driven clusters among Chinese community diabetes populations
Source: J Diabetes. 2024 Aug 13;16(8):e13596. doi: 10.1111/1753-0407.13596 (PMC11320751; doi:10.1111/1753-0407.13596)
Supplement: Supplementary file 2 — Table S1. [file JDB-16-e13596-s002.docx]

**Supplementary Table 1 Characteristics of the clustered variables in Chinese community diabetes populations (when adding PBG to a cluster variable)**

|  | **Total** | **Newcluster1[SIDD]** | **New cluster2[MARD]** | **New cluster3[SOIRD]** | **New cluster4[MIDD]** | **P value** |
| --- | --- | --- | --- | --- | --- | --- |
| **Number (%)** | 6369 | 767(12.04) | 1704(26.75) | 2013(31.61) | 1885(29.60) |  |
| **Age of diagnosis** | 60.62 ±9.11 | 58.57 ±8.02 | 70.15 ±6.41 | 56.47 ±6.86 | 57.28 ±7.33 | <0.001 |
| **BMI (Kg/m2)** | 25.61 ±3.58 | 25.54 ±3.22 | 25.57 ±2.67 | 28.18 ±3.29 | 22.93 ±2.62 | <0.001 |
| **HbA1c (%)** | 6.77 ±1.27 | 9.18 ±1.99 | 6.54 ±0.56 | 6.54 ±0.62 | 6.25 ±0.61 | <0.001 |
| **HOMA-IR** | 2.67 (1.83, 3.90) | 3.48 (2.49, 4.87) | 2.55 (1.98, 3.27) | 3.91 (2.99, 5.19) | 1.62 (1.19, 2.16) | <0.001 |
| **HOMA-β** | 58.09 (37.31, 86.67) | 25.54 (15.68, 37.31) | 62.22 (46.18, 82.63) | 90.94 (67.01, 123.62) | 42.41 (29.27, 58.37) | <0.001 |
| **PBG(mmol/L)** | 11.85 (9.60, 14.15) | 19.10 (16.80, 22.11) | 11.87 (10.20, 13.61) | 11.77 (9.78, 13.47) | 10.90 (7.82, 12.40) | <0.001 |

Note: Continuous variables were expressed as mean ± SD or median (25% quartile, 75% quartile), and categorical variables were presented numerically (proportionally).

Abbreviations: BMI, body mass index; HbA1c, hemoglobin A1c; HOMA -β, homoeostatic model assessment estimates of β-cell function; HOMA-IR, homoeostatic model assessment estimates of insulin resistance, MARD, mild age-related diabetes; MIDD, mild insulin-deficient diabetes; SIDD, severe insulin-deficient diabetes; SOIRD, severe obesity-related and insulin-resistant diabetes.
